# The Mediterranean Diet in Primary and Secondary Prevention of Coronary Heart Disease: Evidence and Mechanisms

**DOI:** 10.3390/nu17223617

**Published:** 2025-11-20

**Authors:** Ewelina Młynarska, Julia Kurcińska, Szymon Kurciński, Weronika Marcinkowska, Klaudia Leszto, Paulina Jakubowska, Jacek Rysz, Beata Franczyk

**Affiliations:** 1 Department of Nephrocardiology, Medical University of Lodz, Ul. Zeromskiego 113, 90-549 Lodz, Poland; 2 Department of Nephrology, Hypertension and Family Medicine, Medical University of Lodz, Ul. Zeromskiego 113, 90-549 Lodz, Poland

**Keywords:** cardiovascular disease, mediterranean diet, prevention, dietary interventions

## Abstract

Cardiovascular disease is a major cause of mortality, and its global reach places it as an object of interest for many researchers worldwide. Steps are being actively taken to combat this disease. Current trends focus on non-invasive prevention, specifically dietary interventions of a relatively easily accessible, modifiable therapeutic option. The most promising seems to be the MD, used in both primary and secondary prevention, as reflected in current research. The bioactive components and mechanisms of this dietary pattern confirm its validity. For now, it is at the forefront of other diets available but needs to be improved in terms of precise guidelines for its application to the individual patient.

## 1. Introduction

The WHO estimates that almost one in three deaths worldwide is due to cardiovascular disease (CVD), with coronary heart disease (CHD) being the leading component [1]. The clinical presentation varies and is dictated, among other things, by the current condition of the vessels supplying blood to the heart muscle. We can distinguish between forms such as stable coronary artery disease (CAD), unstable angina (UA) and myocardial infarction (MI) [2].

In a nutshell, it is a disease that limits blood supply to the myocardium as a result of decreased lumen in the coronary arteries through lipid deposition, smooth muscle proliferation and endothelial dysfunction [3]. This is, in other words, an inflammatory-fibro-proliferative response to vascular endothelial cell (VEC) injury [4]. The vascular endothelium plays an important role in this process, maintaining vascular homeostasis. Therapeutic strategies that prevent endothelial dysfunction and have the potential to slow disease progression include hypoglycemic, statin, anti-inflammatory and antiplatelet drugs [5]. Prevention of heart disease is a wide concept. Cardiovascular risk assessment can be very useful here. For this purpose, the Pooled Cohort Equations risk calculator or the SCORE2 algorithm, for example, are used [6]. In the USA population, risk determinants that tend to increase include old age, diabetes and obesity, while decreasing contributions to pathogenesis are noted in smoking, hypertension, dyslipidaemia and physical inactivity [7]. We can distinguish between primary prevention, which prevents the development of the disease, and secondary prevention, which aims to prevent further cardiovascular episodes in patients with an established diagnosis. Patients diagnosed with CHD are at increased risk of heart attack, stroke and death [8,9]. The causative role of recurrence is not fully understood; so far the influence of genetic predisposition, low HDL cholesterol levels and younger age at the time of the first CAD event has been established [10]. There is evidence that primary and secondary prevention reduces the number of cardiovascular incidents including death [11]. Dietary interventions are becoming increasingly important in the pathogenesis, prevention and treatment of cardiovascular disease due to growing research [12]. The dietary patterns most commonly found in the cardioprotective literature are the Mediterranean diet (MD), Dietary Approaches to Stop Hypertension (DASH) and plant-based diets [13]. Popular in regions of Portugal, Spain, Italy and Greece, MD is associated not only with the prevention of coronary heart disease but also with reduced overall mortality and decreased severity of symptoms of metabolic syndrome, associated with CAD [14].

The aim of this review is to provide an updated and comprehensive overview of recent evidence on the Mediterranean diet’s role in CVD, with a focus on its underlying mechanisms, clinical outcomes, and potential advantages over other cardioprotective dietary patterns.

## 2. Methodology

The objective of this review is to systematically summarize current knowledge on the role of the Mediterranean diet in the primary and secondary prevention of coronary heart disease.

The literature search was conducted between June and August 2025 using the PubMed database, one of the most widely recognized scientific search engines.

The inclusion criteria covered publications from 1998 to 2025, with no geographical restrictions. The search strategy employed the following terms: “Mediterranean diet” AND (“coronary heart disease” OR “coronary artery disease” OR “cardiovascular disease” OR “myocardial infarction”) AND (“primary prevention” OR “secondary prevention”).

Initial articles were selected based on titles and abstracts, and subsequently subjected to a qualitative analysis. Articles that merely mentioned the search terms without discussing the effects of dietary interventions on coronary heart disease, potential pathophysiological mechanisms, or other related aspects were excluded from further consideration.

The review incorporated a variety of publication types, including original research (clinical, cohort, and observational studies), systematic reviews and meta-analyses, narrative review articles, as well as public health guidelines (e.g., WHO, FDA, AHA) and a relevant book chapter to provide context. This comprehensive selection of sources enabled a thorough evaluation of the current evidence.

The findings presented may serve as a foundation for future original research and systematic reviews in this area. This paper provides a descriptive overview of available research findings and is based exclusively on previously published literature, with no new studies involving human or animal subjects conducted for this review.

## 3. Mediterranean Diet

### 3.1. Concept of Mediterranean Diet

**Figure 1:**
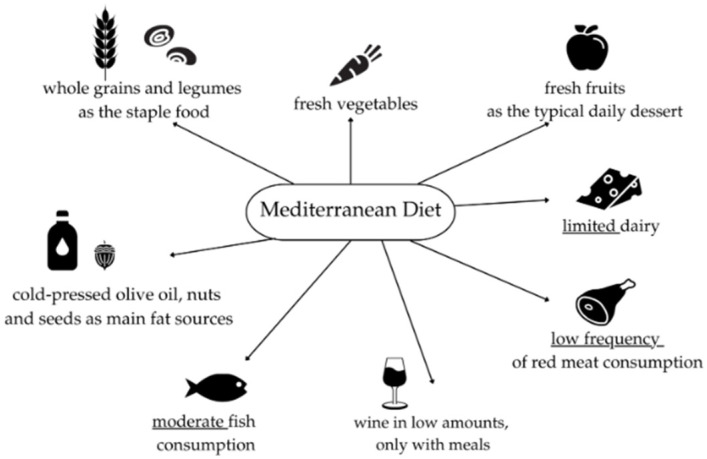
Dietary Characteristics of the Traditional Mediterranean Diet [15,16,17,18]. Figure created using Canva Free (Canva.com, accessed November 2025). All icons used are free elements available under Canva’s Free Content License (https://www.canva.com/policies/content-license-agreement/ (accessed on 1 October 2025)).

The MD denotes the customary dietary pattern historically observed among populations around the Mediterranean basin, although cultural, ethnic, religious and socioeconomic factors produce regional variants [15]. The prototypical MD emphasizes predominantly plant-derived foods, vegetables, fruits, legumes, minimally processed cereals (including bread), potatoes, nuts and seeds, with a preference for seasonal, locally sourced and minimally processed items (Figure 1) [15]. Olive oil (OO), particularly virgin (VOO) and extra-virgin (EVOO), is the primary fat source and is used routinely but not excessively; traditional MD patterns typically keep total fat intake around or below ~30% of energy, with saturated fats usually contributing <8–10% [16,17,18]. Fish and seafood are eaten frequently, while poultry and dairy (notably cheeses and yogurts) are consumed in moderation and red and processed meats are limited. Some traditional MD variants include moderate wine intake, typically red wine with meals, but wine is mentioned only as a cultural element, not as a health recommendation; if consumed, it should be limited to low amounts where culturally appropriate, and alcohol should never be initiated for health reasons because excessive intake is harmful [16,17,18].

### 3.2. Bioactive Components and Mechanisms of the Mediterranean Diet in Cardiovascular Disease

Cardioprotective effects ascribed to the MD likely arise from multiple, overlapping mechanisms rather than a single pathway [18,19,20,21]. Proposed mechanisms include improvements in blood lipid profiles, reduction in systemic inflammation and oxidative stress, enhanced insulin sensitivity, improved endothelial function and decreased platelet aggregation. These outcomes appear to result from a complex mixture of bioactive constituents present in MD foods, including vitamins, glucosinolates (from cruciferous vegetables), minerals, melatonin, phytosterols, carotenoids, a wide array of polyphenols (e.g., resveratrol, hydroxytyrosol), MUFA, PUFA and dietary fiber [18,19,20,21].

Sofi et al. conducted a three-month crossover trial (NCT02641834) comparing an MD to a lacto-ovo-vegetarian diet in overweight adults at low–moderate cardiovascular risk and reported that both dietary patterns produced beneficial changes in cardiovascular risk markers (lipids, glucose, oxidative stress and inflammation) relative to baseline [22].

#### 3.2.1. Lipid Metabolism

A substantial evidence base indicates that MD adherence favorably modifies lipid profiles, a key mechanism for dyslipidemia prevention and atherosclerosis risk reduction [22,23,24]; elevated LDL-C remains a principal modifiable risk factor for atherosclerotic CVD [24,25]. In the Sofi et al. trial the MD yielded greater reductions in triglycerides and more effective regulation of LDL compared with the lacto-ovo-vegetarian pattern [23]. Plant sterols and certain PUFAs (including alpha-linolenic acid, abundant in some nuts) contribute directly to LDL-C lowering [26,27]. Pooled and meta-analytic data on nut consumption show dose-dependent reductions in total and LDL cholesterol with habitual nut intake, with larger absolute reductions in participants with higher baseline LDL or lower BMI and consistent effects across age and sex groups [28,29]. In the PREDIMED randomized primary-prevention trial, MDs supplemented with EVOO or with nuts improved lipid parameters versus a control low-fat diet after short-term intervention [23]; further trials demonstrated that walnut intake reduces not only conventional lipids but also certain atherogenic lipid species (e.g., ceramides and sphingomyelins), suggesting reduced lipotoxicity and improved cardiometabolic profiles [30]. Secondary analyses in PREDIMED indicated that higher baseline plasma ceramide concentrations were associated with increased CVD risk and that assignment to MD intervention arms attenuated the excess risk seen in participants with elevated ceramides [31].

Part of the cholesterol-lowering effect of MDs is attributable to higher dietary fiber typical of traditional patterns: MDs often provide ≈14 g per 1000 kcal, substantially above averages in many industrialized populations. Randomized studies estimate that each additional gram of soluble fiber lowers LDL cholesterol by ~1.12 mg/dL, likely via reduced intestinal reabsorption of bile acids and cholesterol and via short-chain fatty acids produced by microbial fermentation that inhibit hepatic cholesterol synthesis [32,33]. Phytosterols present in nuts, seeds, whole grains, vegetables and fruits further compete with intestinal cholesterol absorption and help reduce plasma cholesterol [33].

#### 3.2.2. Inflammation and Oxidative Stress

**Figure 2:**
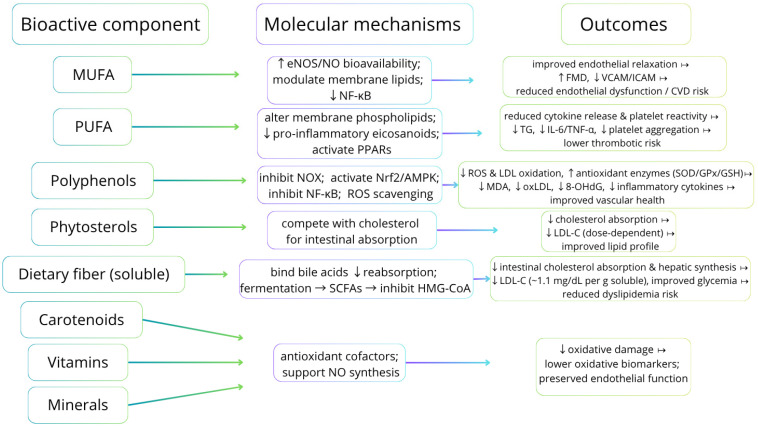
Dietary bioactive components, principal molecular mechanisms, and expected cellular/biomarker/clinical outcomes [16,17,18,19,20,21,22,23,24,25,26,27,28,29,30,31,32,33,34,35,36,37,38,39,40,41,42,43,44,45]. Abbreviations: MUFA—monounsaturated fatty acids; EVOO—extra-virgin olive oil; PUFA—polyunsaturated fatty acids; EPA—eicosapentaenoic acid; DHA—docosahexaenoic acid; ALA—alpha-linolenic acid; eNOS—endothelial nitric oxide synthase; NO—nitric oxide; FMD—flow-mediated dilation; LDL-C—low-density lipoprotein cholesterol; TG—triglycerides; IL-6—interleukin-6; TNF-α—tumor necrosis factor-alpha; NOX—NADPH oxidase; Nrf2—nuclear factor erythroid 2-related factor 2; AMPK—AMP-activated protein kinase; PPARs—peroxisome proliferator-activated receptors; MDA—malondialdehyde; oxLDL—oxidized LDL; 8-OHdG—8-hydroxy-2′-deoxyguanosine; SOD—superoxide dismutase; GPx—glutathione peroxidase; GSH—reduced glutathione; SCFA—short-chain fatty acids; TMA—trimethylamine; TMAO—trimethylamine N-oxide.

Excess generation of reactive oxygen and nitrogen species causes oxidative damage to lipids, proteins and DNA, contributing to CVD pathogenesis and reflected by biomarkers such as malondialdehyde (MDA) and 8-hydroxy-2′-deoxyguanosine (8-OHdG) [34,35]. The MD, rich in polyphenols and in unsaturated fats from EVOO and nuts, modulates pro-oxidant and pro-inflammatory signaling (including NOX, NF-κB, AMPK and Nrf2 pathways), thereby reducing ROS production and inflammatory signaling at the molecular level [36,37]. Clinical and interventional studies generally report that higher MD adherence is associated with reductions in oxidative stress markers (MDA, 8-OHdG and DNA damage assessed by comet assay) and with lower circulating pro-inflammatory cytokines such as IL-6, TNF-α and IL-1β, although findings across trials are heterogeneous and not always statistically significant [38,39,40]. Mechanistic evidence shows that specific MD polyphenols (hydroxytyrosol, oleuropein, resveratrol and flavonoids such as quercetin) inhibit LDL oxidation and NOX activity, activate protective pathways (Nrf2/AMPK) and suppress NF-κB, with concomitant upregulation of endogenous antioxidants (SOD, GPx, GSH) and downregulation of cytokine expression [41]. In populations with metabolic syndrome or obesity, improved inflammatory profiles observed with greater MD adherence are partly mediated by reductions in visceral adiposity and weight loss [42,43,44]. Therefore, a polyphenol-rich MD exerts pleiotropic antioxidant and anti-inflammatory effects that likely contribute to its cardioprotective benefits, while inter-study heterogeneity and variability in biomarker selection highlight the need for standardized long-term trials to establish causality [45] (Figure 2).

## 4. Mediterranean Diet in Primary Prevention of Coronary Heart Disease

CVD, including CHD, are the leading cause of death for both men and women in the general population. Many factors contribute to the development of CHD, such as genetic factors, age, and gender. In addition, modifiable risk factors such as physical activity, smoking and diet are also important. The role of the MD and the effectiveness of educational interventions in primary prevention are particularly emphasized. Many studies confirm that the introduction of MD is associated with improved health and reduced CVD mortality and morbidity [46,47,48,49].

The exact mechanism for the beneficial effects of the MD in reducing the risk of developing CVD is unknown. It is thought that the MD plays a role in lipid downregulation, and protection against oxidative stress, inflammation and platelet aggregation. Prospective observational studies revealed that reduced saturated fat intake is associated with lower plasma cholesterol levels and reduced incidence of CHD, especially when saturated fats are replaced in the diet with polyunsaturated and monounsaturated fats [50]. Nuts, a component of the MD, are a good source of omega-6 and omega-3 fatty acids and plant sterols, and thus have the potential to decrease LDL cholesterol levels and CHD risk. A prospective study showed that eating 5 servings of nuts per week is associated with a significantly lower risk of CHD (RR 0.65, 95% CI 0.47 to 0.89, *p* = 0.0009) [51]. In addition, a randomized study by Solá R. et al., reported that the use of traditional MD promotes lower cardiometabolic risk by decreasing apolipoprotein (Apo)B and the ApoB/ApoA-I ratio, and increasing ApoA-I [52]. Oxidative stress and inflammation play an important role in the pathogenesis of endothelial dysfunction underlying CHD. The MD is related to decreased levels of C-reactive protein (CRP), interleukin-6, markers of endothelial function, and levels of adipocytokines and adiponectin, which are associated with increased CVD risk [47]. Consumption of omega-3 fatty acids is inversely correlated with markers of inflammation and triglyceride levels. Their anti-inflammatory effects presumably involve binding to the G protein-coupled receptor 120 and inhibiting the activity of the NLRP3 inflamasome [53,54]. Moreover, extra virgin olive oil contains olechantal, which inhibits cyclooxygenase (COX), providing protection against platelet aggregation [55].

The ATTICA study conducted in Greece showed that higher adherence to the MD was associated with lower CVD incidence after 5 years of follow-up (*p* < 0.0001). Additionally, an inverse correlation was observed with serum lipid levels, blood pressure, inflammation and coagulation markers associated with CVD, confirming that the MD influences the pathomechanisms underlying CHD [56]. Microsimulation based on the ATTICA study validated the benefit of the MD in primary prevention [57,58]. By improving adherence to MD, at least 851 first CVD events could be averted or delayed [58]. Also, the NHANES III study confirmed that higher dietary adherence was related to a better profile of cardioprotective lipids, glucose metabolism, inflammation and coagulation measurements [59]. Laffond A. et al., analyzed the effects of the MD on mortality and CVD in the general population [14]. In observational studies, they found that higher adherence to the MD was connected to a lower risk of overall mortality from any cause. Furthermore, they showed a reduced risk of CHD and other CVD, such as myocardial infarction and stroke [14]. In assessing the benefits of the MD in the general population, gender-specific studies are also important, given the gender disparities in treatment and prognostic outcomes of CVD. A meta-analysis of 16 prospective cohort studies by Pant A. et al., evaluated the impact of the MD in primary prevention in women [60]. They revealed that higher adherence to the MD was equally beneficial in women and men and was associated with 24% lower CVD incident rates and 23% lower overall mortality. Moreover, such a correlation was also observed for CHD (HR 0.75, 95% CI 0.65 to 0.87; *p* = 0.28) [60]. The Prevención con Dieta Mediterránea (PREDIMED) multicenter trial of primary prevention included 7447 participants at high risk but without current CVD at baseline. The study included men aged 55 to 80 and women aged 60 to 80 and was conducted in Spain with a median follow-up of 4.8 years. General results presented in 2013 showed that a modified MD supplemented with extra-virgin olive oil or nuts was related to a reduced incidence of major cardiovascular events. However, the study was withdrawn due to deviations from the protocol, including the inclusion of household members without randomization and inconsistencies regarding randomization in 2 of the 11 centers [61]. A new report addressing these methodological issues was published in 2018. Compared to a low-fat diet, the PREDIMED intervention was associated with lower rates of major cardiovascular events in primary prevention [61].

In contrast, a meta-analysis by Rees K. et al., including 30 RCTs and seven ongoing trials reported little or no effect of the PREDIMED intervention on CVD mortality (HR 0.81, 95% CI 0.50 to 1.32) or total mortality (HR 1.0, 95% CI 0.81 to 1.24) over 4.8 years [62]. The study also analyzed the effect of the MD on CVD risk factors. There was low-quality evidence of a possible small reduction in total cholesterol and moderate-quality evidence of a reduction in systolic and diastolic blood pressure, with low- or very low-quality evidence of little or no effect on LDL or HDL cholesterol or triglycerides [62].

Recent evidence from the PREDIMED-Plus trial has provided additional insight into the benefits of the MD by incorporating an energy-restricted MD, physical activity promotion, and behavioral support, focusing particularly on weight management and cardiometabolic health. This large, multicenter randomized trial in Spain has shown that the intervention leads to significant weight loss and improvements in metabolic parameters among overweight and obese participants with metabolic syndrome. After 12 months, participants in the intervention group lost an average of 3.2 kg compared to 0.7 kg in the control group, and approximately 34% of the intervention group achieved ≥5% weight loss versus 12% of controls [63]. Moreover, a recent three-year analysis demonstrated sustained reductions in total and visceral adiposity and a slower decline in lean mass, emphasizing the effectiveness of long-term adherence to an energy-restricted MD for obesity and metabolic risk reduction [64]. These findings extend the conclusions of the original PREDIMED study, highlighting that a hypocaloric MD pattern, when combined with lifestyle modification, not only reduces cardiovascular risk but also provides substantial benefits for weight management and metabolic health [65].

## 5. Mediterranean Diet in Secondary Prevention of Coronary Heart Disease

Secondary prevention of CHD includes groups of patients with stable CHD, after acute coronary syndromes, and after revascularization procedures (after PCI or surgical procedures like CABG). Secondary prevention includes therapeutic interventions and patient management that reduce the incidence of complications (including deaths) and recurrences of the disease, as well as inhibit the progression of pathological changes.

There are many factors that influence the occurrence of CHD, some of them are modifiable and the other ones are not. Those we can influence are, for example, lifestyle and diet. When it comes to diet, the Mediterranean diet has recently attracted the most attention. It has a significant role in the primary prevention of CHD, but recently research has focused on its role in secondary prevention. Components of the Mediterranean diet have a cardioprotective effect through bioactive nutrients that act synergistically with the potential to improve glycemia, blood pressure, and lipid profiles in conjunction with anti-inflammatory and antioxidant potential [66,67,68].

The AUStralian MEDiterranean Diet Heart Trial (AUSMED Heart Trial) is a randomized, controlled trial designed to evaluate the effectiveness of the Mediterranean diet in the secondary prevention of CHD. The primary outcome of the AUSMED trial is to evaluate the effectiveness of a 6-month dietary intervention among patients following a Mediterranean diet versus patients on a low-fat diet. The primary outcome is recurrence of cardiac events within 12 months. A secondary outcome is the establishment of clinical biomarkers that may be helpful to explain biological pathways in secondary prevention of cardiac events. Especially changes in status of arterial stiffness, novel immune and inflammatory markers, platelet activity, and body composition, all of this compared to a low-fat diet. The Dietary Inflammatory Index (DII^®^) is a literature-derived dietary score which includes 45 nutritional factors that are known to modulate inflammatory markers, in either a pro- or anti-inflammatory manner [69]. During the analysis of the AUSMED trial, it was shown that the Mediterranean diet has high anti-inflammatory potential, while the low-fat diet has a modest one. A group of CHD patients who followed the Mediterranean diet significantly reduced DII after 6 months. By contrast, the low-fat diet did not influence DII score. Reduction in DII by consumption of the Mediterranean diet showed a tendency to improve the inflammatory marker IL-6, which may be associated with a reduced risk of coronary heart disease progression [70,71].

The CORDIOPREV study (CORonary Diet Intervention with Olive oil and cardiovascular PREVention) is a clinical trial which shows that the Mediterranean diet is also important and effective in secondary prevention. This diet also has additive effect on top of optimal treatment of established cardiovascular disease. CORDIOPREV is a single-blind, randomized trial in 1002 patients with coronary heart disease, who had their last coronary event more than 6 months ago [72]. The primary objective is to compare the effect of two diets: Mediterranean diet and low-fat diet on the incidence of cardiovascular events. The effectiveness of both of these diets was assessed by quantification of intima-media thickness of both common carotid arteries (IMT-CC). The second objective was to investigate the effect on other components related to atherosclerosis progression: carotid plaque number and height. In the carotid artery examination, high-resolution B-mode Doppler ultrasound was used for bilateral assessment of IMT-CC and plaque (number and height), at the beginning of the study and after 5–7 years of dietary intervention [73]. Long-term consumption of a Mediterranean diet, if compared to a low-fat diet, was associated with decreased atherosclerosis progression, as shown by reduced IMT-CC and carotid plaques height. These findings show that the Mediterranean diet is beneficial in the context of secondary cardiovascular prevention [74].

The Hellenic Heart Failure Study was also conducted to evaluate the role of the Mediterranean diet on the prognosis of first-time acute coronary syndrome (ACS) patients. The Hellenic Heart Failure study is a prospective, observational study engaging patients with ACS discharge diagnosis (first or recurrent acute MI or unstable angina). One of the goals was to investigate the interaction between adherence to the Mediterranean diet and left ventricular function on the prognosis of coronary syndrome. The result of this study shows that patients first diagnosed with ACS who followed the Mediterranean diet were protected against recurrent episodes, and have a lower likelihood of post-MI complications and 2-year recurrent coronary events [75]. The results of this study may support the thesis that the Mediterranean diet can be defined as an important addition to the treatment strategy of patients with CHD [76].

Throughout all these studies, patients continued their standard treatment for cardiovascular and other diseases. The use of medications such as lipid-lowering drugs, antidiabetic drugs or antihypertensive drugs was considered to be significant when *p* < 0.05.

The above studies indicate the Mediterranean diet as a promising, non-pharmacological therapeutic method. However, further, more detailed research is needed to fully confirm the role of the Mediterranean diet in the secondary prevention of CHD.

Table 1 summarizes the results and conclusions from various studies about the impact of the Mediterranean diet on the prevention of coronary heart disease.

## 6. Comparison of the Mediterranean Diet with Other Diets

We have found and collected data from studies that reported comparisons of Mediterranean diet versus other diets in CHD. Studies on cardiovascular diseases or cardiovascular risk were also included because coronary heart disease is an important component of these issues [77] (Table 2).

### 6.1. Low-Fat Diet

Giroli et al., in the short-term randomized dietary intervention study compared the change in the whole blood fatty acid profile inducted by MD and Low-Fat Diet (LFD) in patients with CHD [78]. Circulating fatty acids were determined using a rapid analytical method in whole blood. LFD was rich in vegetables, fruit and whole grains, and low in saturated fat (<10% of total caloric intake) and trans unsaturated fats. After the three-month period of diet there was a significant reduction in whole blood saturated fatty acids in both groups, however without difference between diets. Statistically significant difference between groups was identified in four rates: arachidonic acid (augmented with MD but not with LFD; *p* = 0.05), total n − 3 polyunsaturated fatty acids (PUFA) (upgrade in MD and downgrade LFD; *p* = 0.03), eicosapentaenoic acid (EPA) plus docosahexaenoic acid (DHA) (significantly increased in the MD group but without change in the LFD group; *p* = 0.04) and the ratio n − 6/n − 3 polyunsaturated fatty acids (decrease in MD and increase in LFD; *p* = 0.04). Differences related to omega-3 (total n − 3 PUFA and EPA + DHA) may suggest more favorable effect of MD than LFD in patients with CHD [2], because these compounds have a beneficial influence on CHD management [84,85,86,87].

The occurrence of major cardiovascular events (myocardial infarction, revascularisation, ischaemic stroke, peripheral artery disease, and cardiovascular death) in patients with CHD was rated in the CORDIOPREV study-single-center, randomized clinical trial with the median follow-up of 7 years. LFD included less than 30% of total fat (<10% saturated fat, 12–14% monounsaturated fatty acids, and 6–8% polyunsaturated fatty acids), 15% protein, and a minimum of 55% carbohydrates. The efficacy of MD and LFD in secondary cardiovascular prevention has been compared. In the seventh year the researchers registered a total of 198 primary-outcome events: 87 (17.3%) in the Mediterranean diet group of 502 patients and 111 (22.2%) in the low-fat group of 500 patients. The unadjusted HR was 0.745 (95% CI 0.563–0.986) Patients strictly following the recommendations achieved better results in the MD group (HR 0.602, 95% CI 0.385–0.941, *p* = 0·026). Similarly, more beneficial effects of MD than LFD were demonstrated in the evaluation of the heart events (HR 0.745, 95% CI 0.580–0.956, *p* = 0.021). In men, major cardiovascular events occurred in 16.2% in the MD group and 22.8% in the LFD group, with a statistically significant difference in favor of MD (*p* = 0.013). On the contrary, in women no significant difference was found between MD and LFD [79]. Podadera-Herreros et al. compared the effectiveness of MD and LFD in the management of chronic kidney disease in patients with CHD [80]. The researchers assessed kidney function after 5 years of diet use, based on serum creatinine-based estimated glomerular filtration rate (eGFR). A decrease in this indicator was observed in the case of both dietary interventions (*p* < 0.001); however, in the population using MD the decrease in eGFR was significantly lower by 1.58 mL/min/m^2^ (*p* = 0.033). After analyzing subgroups of participants, a significant difference was found among patients with type 2 diabetes, where the decrease in eGFR was lower by 2.07 mL/min/1.73 m^2^ in MD compared to LFD (*p* = 0.040). No significant difference was observed between diets in patients without type 2 diabetes [80].

Yokose et al. compared MD with LFD and low-carbohydrate diet in participants with moderate obesity [81]. In the MD group there were 47.4% patients with CHD. In LFD 32.9% with CHD. The comparison of cardiometabolic risk factors (adiposity, lipid profile, systolic blood pressure fasting plasma insulin concentration) showed the improvement of risk factors in each diet (*p* < 0.05), but no significant differences between diets (*p* > 0.05) [81].

### 6.2. Low Carbohydrate Diet

In the previously mentioned paper by Yokose et al., the low-carbohydrate non–restricted calorie diet was adapted from the Atkins diet [81]. Participants started with an induction phase aimed to provide 20 g of carbohydrates per day for the first 2 months, which was gradually increased to a maximum of 120 g/day for the remainder of the 24-month period without limitation in total calories, protein, or fat. Similarly to LFD and MD, research results indicated the improvement in cardiometabolic risk parameters (adiposity, lipid profile, systolic blood pressure fasting plasma insulin concentration) during adopting the individual diets (*p* < 0.05), but with no significant difference between groups [81].

### 6.3. Lacto-Ovo-Vegetarian Diet

Typical lacto-ovo vegetarian diet (VD) is characterized by abstinence from consuming meat and meat products, poultry, fish and seafood, and flesh from any other animal, but including eggs and dairy products [83]. Significant risk reduction in CAD mortality associated with VD and beneficial effect on CAD risk factors have been demonstrated [88,89,90]. We have not found any manuscript comparing MD and VD in patients with CAD; nevertheless, some studies assessed the impact of those two diets on cardiovascular risk markers.

The CARDIVEG Study was the randomized controlled trial assessing the effect of VD compared with MD after 3 months of dietary intervention in overweight patients with a low-to-moderate cardiovascular risk profile. The researchers targeted the primary outcomes as the differences in total body weight, BMI and fat mass. The secondary outcomes were differences in changes in all the circulating cardiovascular risk parameters. The primary outcomes showed equally effective results in both diets in all parameters, without significant differences between groups (respectively, for VD and MD: body weight reduction −1.88 kg and −1.77 kg; BMI reduction −0.64 kg/m^2^ and –0.67 kg/m^2^; fat mass reduction −1.23 kg and 1.46 kg). In the VD group there was significant decrease in LDL-cholesterol levels (−5.44%; no significant change in MD) uric acid levels (−2.89%; no significant change in MD) and vitamin B12 levels (−5.06%; no significant change in MD). In the triglyceride levels there was a significant decrease in MD group (−5.91%), with non-significant change in VD. No differences between diets have been noted in the oxidative stress profile. Only interleukin-17 levels decreased significantly in the inflammatory profile. There was a significant decrease (by 36.3%) in the MD phase and an increase (by 37.57%) in the VD phase of this pro-inflammatory interleukin levels. To sum up, both groups reached a significant improvement in cardiovascular risk profile. A total of 44.2% participants in the VD group and 34% in the MD group modified their risk category by reaching the target values determined by the European Society of Cardiology [22]. Although these data do not directly relate to CHD, further analysis of the obtained results may allow us to assess the impact of VD and MD in the prevention of CHD.

Ronca et al. analyzed the data from CARDIVEG study and assumed as a primary outcome the HDL-cholesterol efflux capacity (HDL-CEC), which can be a better cardiovascular risk maker and CHD predictor than HDL level [82,91]. HDL-CEC may be inversely related to the incidence of cardiovascular risk and some studies reported about the relationship between macrophage-specific HDL-CEC and coronary events [92,93,94,95]. More beneficial impact of MD was noted in HDL-CEC mediated parameters (total efflux: 22.24 and 20.24 for MD and VD, respectively; *p* < 0.001), so the authors considered the MD as a better choice for cardiovascular prevention than VD [82]. Cesari et al. also used data from CARDIVEG, taking the level of circulating progenitor cells (CPCs) as a reference point [83]. These cells were identified as a prospective biomarker of future cardiovascular events [22]. They are attributed to important roles in endothelial regulation, and they are probably acting through inflammatory modulation. Three types of CPCs were examined. A significant decrease in all three groups was observed after VD intervention (*p* < 0.05). On the other hand, MD intervention led to a significant increase in CPCs in all groups (*p* < 0.05), so that MD, but not VD, may positively moderate CPCs for cardiovascular prevention [96]. All of the mentioned studies were directly comparing MD with other types of diets. Different parameters in various patient groups have been studied. Not only CHD prevention and management indicators, but also cardiovascular risk markers have been analyzed. Despite the wide range of research and diverse methodology, the benefits of MD can be seen. In many cases, the results indicated greater benefits of MD than other diets. It is therefore likely that MD may be the right choice of nutritional model for CHD prevention.

## 7. Conclusions

Current evidence supports the Mediterranean diet as an effective, evidence-based non-pharmacological strategy for primary and secondary prevention of coronary heart disease, with large, randomized trials (PREDIMED) and the CORDIOPREV trial demonstrating reductions in major cardiovascular events or atherosclerosis progression. Mechanistic and translational data indicate that key components of the pattern, notably polyphenols, monounsaturated fats and omega-3 PUFAs, modulate oxidative- and inflammation-related pathways (NOX, NF-κB, AMPK, Nrf2) and reduce LDL oxidation. They also augment endogenous antioxidant defenses and attenuate adipose-derived cytokine production, providing biological plausibility for cardioprotection. Nevertheless, substantial heterogeneity in trial designs, variable biomarker selection and inconsistent effect sizes limit causal inference and highlight the need for standardized endpoints and longer follow-up. The impact of pharmacotherapy used by patients, especially in secondary prevention, may be questionable. In the future, studies should focus on ensuring that all participants in both the research and control groups will be consistent and uniform in medication use, to best demonstrate the impact of dietary management. Further research into the etiopathogenesis and risk factors of coronary artery disease may enable development of new therapies; meanwhile, prevention (including diet) and early diagnosis remain the primary management strategies. Future research should therefore prioritize well-powered, mechanistically informed randomized trials with harmonized inflammatory/oxidative biomarkers, integration of metabolic and behavioral phenotyping, and evaluation of implementation strategies to define optimal, personalized Mediterranean-based prescriptions, develop predictive and personalized guidelines, and ensure sustained adherence across diverse clinical populations. In clinical practice, clinician-tailored promotion of the Mediterranean dietary pattern, adapted to patient preferences, comorbidities and cultural context, represents a pragmatic, evidence-based adjunct to conventional risk-reduction therapies for coronary heart disease.

## Figures and Tables

**Table 1:** Summary of results and conclusions from various studies about the impact of the Mediterranean diet on the prevention of coronary heart disease.

	Study	Conclusions
Primary Prevention	ATTICA	High adherence to the MD is associated with lower CVD incidence. MD has a beneficial effect on the pathomechanisms underlying CHD [56,57,58].
	NHANES II	DM adherence is related to a better profile of cardioprotective lipids, glucose metabolism, inflammation and coagulation parameters [59].
	PREDIMED	Modified MD supplemented with extra virgin olive oil or nuts reduces the incidence of cardiovascular events with better results than a low-fat diet [61,62].
	PREDIMED-Plus	Hypocaloric MD combined with lifestyle modification reduces cardiovascular risk and improves weight management and metabolic health [63,64,65].
Secondary Prevention	AUSMED	MD has a high anti-inflammatory potential and significantly reduces inflammatory markers in patients with CHD [69,70,71].
	CORDIOPREV	MD decreases the progression of atherosclerosis in patients with CHD [72,73,74].
	Hellenic Heart Failure Study	MD in patients after first diagnosed acute coronary syndrome protects against recurrent episodes [75,76].

**Table 2:** Diets compared to Mediterranean diet in prevention and management of coronary heart disease or cardiovascular risk [22,75,76,77,78,79,80,81,82,83].

Diet Compared to MD	Paper	Patients	Primary Outcomes	Results	Selected Assessment Tools
Low fat diet	Giroli et al.	patients with CHD	blood fatty acids profile	improvement in both, advantage of MD in omega-3	MeDAS (Mediterranean Diet Adherence Screener), plasma fatty acid levels measured using gas chromatography (GC)
	Delgado-Lisa et al.	patients with CHD	major cardiovascular events	improvement in both, more beneficial in MD	Food frequency questionnaire (FFQ), 14-item MedDiet questionnaire, 9-item Low Fat Diet questionnaire
	Podadera-Herreros et al.	patients with CHD	kidney function in CHD (eGFR measurement)	significantly lower decrease in eGFR in MD	
	Yokose et al.	patients with moderate obesity; in MD group 47.4% with CHD, in LFD 32.9% with CHD	cardiometabolic risk factors (adiposity, lipid profile, systolic blood pressure fasting plasma insulin concentration)	improvement in both, no significant differences between diets	DXA scan Uricase assay (Roche Modular P)
Low carbohydrate diet	Yokose et al.	patients with moderate obesity; in MD group 47.4% with CHD, in LCD 28.4% with CHD	cardiometabolic risk factors (adiposity, lipid profile, systolic blood pressure fasting plasma insulin concentration	improvement in both, no significant differences between diets	Uricase assay (Roche Modular P)
Lacto-ovo vegetarian diet	Sofi et al.	overweight patients with a low-to-moderate cardiovascular risk profile	total body weight, BMI, fat mass	↓ in both, no significant differences between diets	Assessment used body and composition measures stadiometer, Tanita TBF-410 bioelectrical impedance analyzer dietary intake via the National Health and Nutrition Examination Survey food questionnaire
			LDL-cholesterol, uric acid, vitamin B12 levels	↓ in VD, no significant changes in MD	
			Triglyceride levels	↓ in MD, no significant changes in VD	
			Oxidative stress profile	no significant differences between diets	
			IL-17 level	↓ in MD, ↑ in VD	
	Ronca et al.	overweight patients with a low-to-moderate cardiovascular risk profile	HDL-cholesterol efflux capacity	MD > VD	
	Cesari et al.	overweight patients with a low-to-moderate cardiovascular risk profile	level of circulating progenitor cells	↑ in MD, ↓ in VD	a modified version of the National Health and Nutrition Examination Survey food questionnaire-compliance

Abbreviations: CHD—coronary heart disease; LCD—low carbohydrate diet; LFD—low fat diet; MD—mediterranean diet; VD—lacto-ovo vegetarian diet; DXA (Dual-energy X-ray Absorptiometry); ↓—decrease; ↑—increase.

## Data Availability

No new data were created or analyzed in this study.

## Abbreviations

The following abbreviations are used in this manuscript:

8-OHdG	8-hydroksy-2′-deoxyguanosine
ACS	Acute cronary syndrome
ALA	Alpha-linolenic acid
AMPK	AMP-activated protein kinase
Apo	Apolipoprotein
BMI	Body mass index
CABG	Coronary artery bypass grafting
CAD	Coronary artery disease
CHD	Coronary heart disease
CI	Confidence interval
COX	Cyclooxygenase
CPCs	Circulating progenitor cells
CRP	C-reactive protein
CVD	Cardiovascular disease
DASH	Dietary Approaches to Stop Hipertension
DHA	Docosahexaenoic acid
eGFR	Estimated glomerular filtration rate
EPA	Eicosapentaenoic acid
EVOO	Extra virgin olive oil
GPx	Glutathione peroxidase
GSH	Glutathione
HDL	High density lipoprotein
HDL-CEC	High density lipoprotein-cholesterol efflux capacity
HR	Hazard ratio
IL	Interleukin
IMT-CC	Intima-media thickness of both common carotid arteries
LCD	Low carbohydrate diet
LFD	Low fat diet
LDL-C	Low density liporotein-cholesterol
MDA	Malondialdehyde
MD	Mediterranean diet
MI	Myocardial infraction
MUFA	Monounsaturated fatty acids
NF	Nuclear factor
NLRP3	NOD-like receptor protein 3
NOX	Nitric oxide metabolites
Nrf2	Nuclear factor erythroid 2-related factor 2
OO	Olive oil
PCI	Percutaneous coronary intervention
PUFA	Polyunsaturated fatty acids
ROS	Reactive oxygen species
SOD	Superoxide dismutase
TNF	Tumor necrosis factor
UA	Unstable angina
VD	Vegetarian diet
VEC	Vascular endothelial cell
VOO	Virgin olive oil
WHO	World Health Organization
